# An empirical study on the impact of charitable medical care on residents’ health level

**DOI:** 10.3389/fpubh.2025.1591503

**Published:** 2025-05-30

**Authors:** Boxun Han, Qilin Zhang

**Affiliations:** Center for Social Security Studies, Wuhan University, Wuhan, China

**Keywords:** charitable medical care, health level, accessibility of medical services, quality of medical services, multilevel regression model

## Abstract

**Introduction:**

Health is a crucial foundation for personal growth and the realization of a better life. As an organic part of China’s multitiered medical security system, charitable medical care has an important regulatory effect on the health level of residents. However, the improvement in residents’ health level by charitable medical care remains to be validated.

**Methods:**

Based on the panel data of the China Family Panel Studies (CFPS 2012–2020), this study establishes an evaluation index system for residents’ health level, empirically analyses the impact of charitable medical care on residents’ health level, and explores in-depth the transmission mechanism therein.

**Results:**

This research shows: First, an increase in the level of charitable medical donations can significantly improve the health level of residents. After conducting strict robustness tests, this conclusion still holds. Second, the accessibility and quality of medical services are the paths through which charitable medical care affects residents’ health level, and both play a mediating role. Finally, in the heterogeneity test, for groups of residents with higher levels of trust, lower annual household income, and higher total medical expenses, charitable medical care has a stronger promoting effect on their health level.

**Discussion:**

These research results provide empirical evidence and policy implications for the optimization and reform of China’s medical security system in the context of the Healthy China Strategy.

## 1 Introduction and literature review

The pursuit of health is a shared vision of people worldwide. Individual health serves as the cornerstone of survival and development, whereas the health of people serves as the foundation for a country’s prosperity. With the acceleration of China’s industrialization, urbanization, and population ageing, the disease burden has been continuously increasing. Chronic non communicable diseases such as cardiovascular and cerebrovascular diseases, cancer, and chronic respiratory diseases account for 88% of the total number of deaths and over 70% of the total disease burden (1). The prevention and control of major infectious diseases, such as certain types of cancer, tuberculosis, and AIDS, remain severe challenges. In 2015, to address these issues, the Chinese government proposed the implementation of the “Healthy China” development strategy and formulated and implemented the “Outline of the Healthy China 2030 Plan,” which serves as an action plan for promoting the construction of Healthy China in the next 15 years.^1^ The report of the 20th National Congress of the Communist Party of China^2^ also clearly stated, “Promote the construction of Healthy China. Place the protection of people’s health in a strategic position of priority development and improve policies for promoting people’s health.” Therefore, against the backdrop of the Chinese government’s efforts to promote the “Healthy China” initiative, how to make full use of medical and health resources to promote the health of the entire nation has become a hot topic.

Health is an essential part of human welfare and living standards (2). With the continuous development and progress of the economy and society, the health of residents has increasingly become a crucial metric for achieving high-quality population development (3). Medical and health undertakings serve as vital pillars for safeguarding residents’ health and family wellbeing. The quality and accessibility of medical services directly influence the quality of life and sense of happiness of residents (4). However, currently, China’s medical and health system is still in the stage of increasing reform. Prominent issues such as uneven medical resource distribution, high medical costs, and an imperfect medical system persist. These problems are particularly acute among underdeveloped regions in central and western China, rural residents, vulnerable social groups, low-income families, and patients with major diseases (5, 6). According to relevant data,^3^ the persistently high out-of-pocket medical expenses of Chinese residents at present are a practical problem that urgently needs to be resolved. The proportion of residents’ medical expenditures in total consumption expenditures rose from 6.7% in 2002 to 9.2% in 2023. More than 70% of Chinese residents have a reimbursement rate within the scope of medical insurance of no more than 70%. Among hospitalized patients, 24.2% abandoned hospitalization because of financial difficulties. Against this backdrop, to attain the strategic goal of “joint construction, shared benefits, and universal health” for Healthy China, charitable medical care, as a social mechanism for pooling private resources (7), is gradually emerging as an important complementary force for public health undertakings and plays an indispensable role in improving the quality of medical services and enhancing the health level of residents.

Charitable medical care not only embodies the humanitarian spirit and social responsibility but also helps alleviate problems such as shortages of medical resources and insufficient accessibility of medical services. Especially in poor and underdeveloped regions, the role of charitable medical care is especially significant. Specifically, charitable medical care is a social supplementary mechanism based on voluntary donations. It follows the principles of charity and mutual assistance and pools social forces through charitable acts such as free donations and aims to address the medical problems of those in need (8, 9). At present, the main recipients of charitable medical care in China are low-income groups, expenditure-induced poverty groups, special-hardship groups, and specific assistance projects groups. In terms of the implementation paths of charitable medical care, the participation of multiple entities, such as charitable organizations, government departments, enterprises, and medical institutions, presents diverse features. On the one hand, the most common form of charitable medical care is charitable donation projects involving tangible resources such as funds, medicines, and medical devices. On the other hand, charitable medical care can also provide intangible resources such as free medical consultations, policy publicity, and talent and technology for specific groups or specific diseases. Some research suggests that current charitable donations, mainly in the form of donations of money and goods, are the main means of implementing charitable medical care in China. They are characterized by flexibility and convenience and provide beneficiaries with maximum freedom in use (10). In the Chinese context, the reason charitable donations have become a central component of charitable healthcare is deeply intertwined with the characteristics of China’s medical security system, the philanthropic landscape, and practical needs. First, while China’s basic medical insurance covers over 1.3 billion people, significant out-of-pocket expenses persist for critical illnesses, rare diseases, and chronic conditions. The Charity Law explicitly classifies medical assistance as a form of statutory philanthropy, granting legal status to monetary donations. Second, medical resources in China are unevenly distributed, concentrated primarily in eastern coastal regions. Monetary donations can overcome geographical barriers through a mechanism of “national planning and targeted disbursement”—a capability for cross-regional resource allocation that forms such as volunteer services struggle to match. Third, China’s pragmatism-oriented donation culture leads residents to prefer charitable acts with “visible impacts.” Compared to abstract services like health advocacy or psychological counseling, donating money or goods is a more tangible form of giving, making it easier to earn public recognition.

Moreover, in recent years, with the rapid development of digital transformation and information technology, the scale and influence of charitable medical care in China have been continuously expanding, and an increasing number of individuals and organizations have participated in charitable medical care. According to the statistics in the “China Social Public Welfare and Charity Guide: Medical and Health”,^4^ nearly one-third of China’s public welfare and charity funds pour into the education field, whereas the medical and health field closely follows, with a proportion of 27%. However, if all public welfare projects related to medical and health are considered, the gap between the two will further narrow or even reverse. In addition, in recent years, online personal fundraising platforms for serious diseases in China, such as Shuidichou and Qingsongchou,^5^ have become important donation channels for charitable medical care. The amount of donations raised each year exceeds 10 billion yuan. As of 2021, approximately 6 million families have successfully raised charitable medical donations (11). Thus, charitable medical care is crucial for addressing the practical shortcomings of insufficient medical security supply in China. Based on the above, this study suggests that in China, charitable donations are the main form of realizing charitable medical care and the core content of internet charity in the digital age. Therefore, this paper focuses on the empirical effect of charitable medical donations on residents’ health. Notably, this study does not dismiss other forms of charitable healthcare but rather highlights the foundational role, adaptability, and scale advantages of monetary and in-kind donations in the specific Chinese context.

At present, the academic community has conducted extensive and in-depth discussions on the factors influencing residents’ health. Research suggests that diverse factors, such as education, socioeconomic status, retirement, income level, and medical and health resources, significantly affect residents’ health (12–16). Among them, most scholars agree that the development of medical and health undertakings is a key guarantee for improving residents’ health level and alleviating health inequality (17, 18). Some research indicates that people living in cities with higher health care expenditures exhibit better health levels (19). In other words, an increase in public health spending helps meet people’s medical needs and safeguards residents’ physical health (20). Therefore, ensuring the fairness of medical and health services is one of the top priorities in any health care system (21). However, some scholars have reported that there is relatively serious unfairness in the allocation of medical resources in China among urban and rural areas, regions, and social strata (22) and that there are inequalities in the accessibility of residents’ utilization of medical services (23). To address this, some scholars have proposed accelerating the development of charity-based medical care, giving full play to the supporting role of charity-based medical care in the public health system, and weaving a tight health safety net for residents (24). The government should strengthen cooperation and coordination with charity-based medical care and promptly promote the connection and integration of charity-based medical care with China’s multilevel medical security system (25, 26). Moreover, it is necessary to combine China’s national conditions and learn from the advanced experiences of countries with relatively developed charitable medical care, such as the UK and the US (27–29).

In the relevant research on charitable medical care, some scholars have expounded on the potential of modern financial charitable tools in the medical and health fields (30), summarized the key factors for the success of charitable medical activities by using systematic reviews (31), conducted in-depth analysis of typical charitable projects by comprehensively applying research methods such as historical review and case analysis, and summarized the operation models and development directions of charitable medical care (32, 33). In addition, some scholars have also carried out empirical research on charitable medical care. Waite (34) explored the role of charity in public health services and analyzed inequality in the receipt of charitable resource support by NHS trusts in England. Some studies have used medical crowdfunding projects on the Tencent Charity Platform, a well-known medical crowdfunding platform in China, to explore the impact of their text features on the success rate (35). Some scholars have expressed concerns about possible fraud in charitable medical care. Charitable medical care may expose participants to significant privacy risks and instead widen the income gap and health inequality (36, 37). In general, current research on charitable medical care is relatively weak, and research on the impact of charitable medical care on residents’ health is even rarer. Few studies have analyzed the impact effect and mechanism of charitable medical care on residents’ health from an empirical perspective, which is not conducive to understanding the value connotation and functional positioning of charitable medical care in academic and practical circles. This study uses panel data from the China Family Panel Studies (CFPS) to empirically analyze and test the relationship between charitable medical care and residents’ health and reveal the internal logical chain between them.

The contributions of this paper are as follows: First, unlike previous qualitative studies that focused on the macrolevel and theoretical aspects, this paper demonstrates the impact of charitable medical care on residents’ health from an empirical perspective, filling a gap in the relevant research field. Second, it analyses the transmission mechanism between charitable medical care and residents’ health in detail, demonstrates the mediating roles played by the accessibility of medical services and the quality of medical services, clarifies the mechanism of action of charitable medical care, and enriches the research on charitable medical care. Third, the research of this paper provides an empirical basis for the practical community to accelerate the development of charitable medical care, which is conducive to promoting the high-quality development of the charity cause and simultaneously facilitating the realization of China’s “Healthy China” strategic goal.

## 2 Theoretical foundations and research hypotheses

### 2.1 Charitable medical care and residents’ health

Against the backdrop of advancing the Healthy China Initiative and continuous improvements to the social healthcare security system, charitable healthcare—an important complement to China’s social security system—plays a crucial role in enhancing residents’ health outcomes. This study will draw on social support theory and the health belief model to analyze the topic from two perspectives: the external environment and individual psychological perceptions.

On the one hand, since the 1970s, the concept of social support has gradually expanded from the field of psychology to the field of sociology and has been continuously refined and enriched thereafter. Social support can be simply understood as any resources flowing in and out within social relationships (38). Many definitions and interpretations of social support can be found in the literature. The academic community has not yet reached a consensus, but there are common characteristics among them. Scholars from various countries generally believe that social support refers to the voluntary and gratuitous relief or assistance provided to socially vulnerable groups through the inflow and outflow of material and spiritual resources within the social network (39, 40). In the social support system, the subject, the object, and the content of support jointly constitute the organic elements of the system. The subject is the provider of social support, the object is the recipient of social support, and the content of support can be divided into objective and tangible material support, as well as subjective and intangible spiritual support, according to specific circumstances (41).

The social support theory emphasizes the various forms of help and support that assisted individuals in their social network, including material assistance and emotional comfort, which have a significant impact on an individual’s physical and mental health. As a special form of social support, charitable medical care has had markedly positive effects on residents’ health through the provision of medical assistance, health education, spiritual consolation, and other forms of support. First, for many low-income people or those in remote and underdeveloped areas, medical expenses are often a heavy burden. Moreover, due to the scarcity of medical resources, these patients may be unable to obtain timely and effective medical services. In the context of social support theory, charitable medical care can provide them with tangible support, namely, material or financial assistance, relieve their economic pressure, and provide essential medical resources, thus ensuring their basic life safety and health needs. Second, illness not only affects residents’ physical health but also may cause considerable psychological stress and negative emotions. The funds donated for charitable medical care can be used to provide intangible support, that is, focus on the mental state of patients. Through means such as psychological counseling, it helps patients relieve negative emotions such as anxiety and fear. For patients with chronic or serious illnesses, prolonged illness-related distress can easily lead to psychological issues. Charitable healthcare’s psychological interventions effectively improve patients’ mental states, enhance their psychological resilience, and promote overall physical and mental health. Finally, charitable healthcare initiatives bring together diverse stakeholders—including the government, enterprises, social organizations, and volunteers—building a broad social network for residents. When receiving charitable healthcare, patients establish connections with these stakeholders, enabling them to access more social resources and information. The expansion of social networks not only enriches residents’ access to resources but also strengthens their sense of social belonging, producing a positive impact on health.

On the other hand, the Health Belief Model (HBM), first proposed by Hochbaum in 1958 (42), is a pivotal theoretical framework in social psychology used to explain and predict individual health behaviors. Designed to explore why people adopt or reject health-related actions, it also guides interventions aimed at shaping health behaviors. The model posits that individual health decisions are not purely rational but arise from a combination of subjective perceptions and external factors (43). Later developed and refined by social psychologists such as Becker and Rosenstock through their research (44), its core components now include disease susceptibility, disease severity, perceived benefits of action, perceived barriers to action, and self-efficacy (45). First, charitable healthcare enhances residents’ perception of disease threats. Along with providing medical assistance, it typically incorporates health education initiatives. Through health lectures, educational materials, and other outreach methods, residents gain knowledge about disease mechanisms, symptoms, risks, and prevention strategies. This clarity about diseases’ susceptibility and severity strengthens their awareness of the need for prevention. Second, charitable healthcare boosts residents’ confidence in behavioral change. By offering professional medical advice and personalized health guidance, it helps individuals understand how to adopt health behaviors to prevent and manage diseases. When residents observe themselves or others successfully improving health by following these recommendations, their self-efficacy in behavioral change increases significantly. Finally, charitable healthcare reduces barriers to behavioral change. Economically, it alleviates medical costs through fee waivers and subsidies, easing the financial burden of seeking care. In terms of service accessibility, mobile clinics and telemedicine address challenges faced by residents in remote areas. Regarding information access, it provides comprehensive and accurate health and medical resource information, ensuring individuals do not miss optimal treatment or adopt harmful behaviors due to information asymmetry.

Therefore, this paper proposes the following hypotheses:

*H1:* The implementation of charitable medical care has a significant positive effect on the health of residents.

### 2.2 Transmission mechanism: accessibility and quality of medical services

As mentioned above, as a social support mechanism aimed at helping economically disadvantaged groups or vulnerable groups access necessary medical services, charitable medical care has a no negligible effect on residents’ health. Some research indicates that the accessibility and quality level of health care services are two key factors influencing residents’ health conditions: measuring consumer satisfaction and evaluating the performance of the health care system (46, 47). Therefore, as an important part of China’s multilevel medical security system, charitable medical care, in terms of improving residents’ health, not only addresses the issue of the availability of medical services but also gradually solves the problem of the quality of medical services. According to Social Capital Theory, social networks serve as critical channels for resource allocation (48). During operation, charitable healthcare requires broad mobilization of societal actors, including government agencies, enterprises, medical institutions, charitable organizations, and volunteers. These stakeholders form complex social networks through collaboration, within which resources are allocated and integrated based on established norms and trust relationships (49). Social Capital Theory effectively explains this process of resource integration, illustrating how charitable healthcare achieves efficient resource allocation through social networks to enhance access to and the quality of medical services.

#### 2.2.1 Accessibility of medical services

The accessibility of medical services refers to the ease with which residents can obtain qualified medical and health resources, including price acceptability, transportation convenience, resource availability, subjective acceptability, and matching of medical service supply and demand (50). Among them, the factors that have the most fundamental impact on the health level of residents can be summarized as geographical accessibility and economic accessibility (51). Drawing on Social Capital Theory, charitable organizations build extensive social networks with multiple stakeholders to integrate resources, enabling medical services to reach broader regions—especially underserved remote areas such as rural villages and poverty-stricken regions, as well as vulnerable populations like rare disease patients. By setting up or subsidizing primary medical institutions and carrying out free medical consultation services, charitable medical care can incorporate these areas and groups into the medical service network. This not only reduces the gap in medical resources between urban and rural areas and among different regions but also significantly enhances the geographical accessibility of medical services in these areas and for these groups. On the other hand, charitable organizations act as information bridges within social networks (52), appropriately allocating resources to residents in need and significantly reducing barriers to accessing medical care. For groups that cannot access necessary medical services for economic reasons, charitable medical projects usually reduce the economic burden on patients and their households and lower medical costs for patients through financial assistance and fee reduction. This enables these groups to receive timely treatment. This not only improves the economic accessibility of medical services but also promotes the realization of medical fairness, ensuring that medical resources can benefit a wider range of social groups.

#### 2.2.2 Quality of medical services

The quality of medical services pertains to the professional acumen and service quality manifested by medical institutions and medical staff when providing medical services. It encompasses multiple aspects, such as the advancement of medical technology, the perfection of medical equipment, the professional competence of medical staff, and their service attitudes (53). An increase in the quality of medical services implies that patients are entitled to higher-quality and more efficient medical services, which helps to enhance the treatment effect and the speed of recovery. Drawing on Social Capital Theory, charitable healthcare leverages extensive social connections and influence to attract and mobilize substantial professional medical resources (54). It increases investment in medical resources and improves healthcare facility conditions through charitable medical donations. Charitable medical projects can donate advanced medical equipment, medicines, and consumables to relevant areas, especially remote areas with scarce medical resources. The investment and application of these medical resources not only improve the accuracy of medical diagnosis and the effectiveness of treatment but also promote the standardization and regularization of medical services, enabling patients to obtain high-quality medical services without having to travel long distances. On the other hand, charitable medical donations can contribute to the cultivation of medical talent and the innovation of medical technology. Charitable healthcare programs leverage their social networks and resources to attract and bring together many medical professionals (55), organizing various medical academic exchange activities. These activities not only enable grassroots healthcare workers to keep up with cutting-edge medical knowledge and new technologies but also provide them with a platform to share experiences with peers. Through training, healthcare workers can master new diagnostic and treatment methods, enhancing their professional competence and delivering higher-quality, more advanced medical services to residents.

In summary, this paper proposes the following hypotheses:

*H2:* Accessibility of medical services is the path through which charitable medical care affects residents' health. That is, the development of charitable medical care will improve the accessibility of medical services, thereby promoting an improvement in the health of residents. The transmission path is as follows: "Charitable Medical Care → Accessibility of Medical Services → Residents' Health".

*H3:* The quality of medical services is the path through which charitable medical care affects residents' health. That is, the development of charitable medical care promotes an improvement in the quality of medical services, thereby promoting an improvement in the health level of residents. The transmission path is as follows: "Charitable Medical Care → Quality of Medical Services → Residents' Health".

## 3 Data sources, variable selection and model construction

### 3.1 Data sources

The data used in this paper are sourced from panel data from the China Family Panel Studies (CFPS) from 2012, 2014, 2016, 2018 and 2020. The CFPS^6^ aims to track and collect data at the individual, family and community levels; reflect changes in China’s society, economy, population, education and health; and provide a data foundation for academic research and public policy analysis. Owing to the significant differences in health evaluation criteria between children and adults, to ensure the rationality of sample selection, this paper excludes sample groups under 18 years of age. Moreover, samples with severely missing relevant data were also excluded. Eventually, a total of 49,593 observations are retained in this paper. The relevant data on the donation amounts of charitable medical care in various provinces in different years are sourced from the China Statistical Yearbook, the China Charity Development Report, the China Civil Affairs Statistical Yearbook, as well as the statistical yearbooks of various provinces, autonomous regions and municipalities directly under the Central Government. The data related to the provincial control variables are sourced from the China Stock Market and Accounting Research Database (CSMAR).^7^ In addition, to avoid the impact of extreme data on the analysis results, this paper conducts a winsorization treatment on all continuous variables at the 1% level at both the upper and lower ends.

### 3.2 Variable selection

#### 3.2.1 Dependent variable

The dependent variable in this paper is residents’ health. Unlike previous studies, to construct a scientific and reasonable evaluation index system for residents’ health, this paper refers to the Short Form-36 Health Survey (SF-36) and constructs a comprehensive health evaluation system that includes overall health (Health), physical health (P-Health), mental health (M-Health), and self-rated health (S-Health). Among them, for physical health, three secondary indicators are selected, namely, the BMI, disease condition, and independence ability. For mental health, two secondary indicators are selected, namely, mental state and attitudes toward life. The entropy weight method is used to calculate the corresponding weight coefficients and scores of each indicator. The weight and score of overall health are the sum of the weights and scores of the three indicators of physical health, mental health, and self-rated health, which is a comprehensive indicator.^8^ The specific settings of each indicator are shown in Table 1.

**Table 1:** Evaluation index system for residents’ health.

First - level indicators	Second - level indicators	Indicator connotation	Weight coefficient
S-Health	Self - assessment score	Based on the responses to the question “How do you rate your health?” in the CFPS questionnaire, 1 represents “very healthy,” 2 represents “quite healthy,” 3 represents “fairly healthy,” 4 represents “average,” and 5 represents “unhealthy.”	0.337
P-Health	BMI	The Body Mass Index (BMI), simply referred to as the physique index, is an internationally commonly used standard for measuring a person’s degree of fatness or thinness and their health status. The calculation formula is: BMI = weight (kg) divided by the square of height (m). A BMI value within the range of 18.5–23.9 is considered the normal range, in which case the value is set to 0. Otherwise, the value is set to 1.	0.004
	Disease condition	Based on the responses to the question “Have you had any chronic diseases in the past six months?” in the CFPS questionnaire, 1 represents “yes” and 0 represents “no.”	0.213
	Independent ability	Based on the responses to the seven questions in the CFPS questionnaire, namely, “Can you carry out outdoor activities, have meals, engage in kitchen activities, use public transportation, go shopping, do cleaning, and do laundry independently?,” 1 represents “no” and 0 represents “yes.” Then, sum the values of these seven questions.	0.008
M-Health^1^	Mental state	Based on the responses to the six questions in the CFPS questionnaire, namely, “Do you feel depressed, find it difficult to do anything, have trouble sleeping, feel lonely, feel sad, or think life cannot go on?,” for each question, 1 represents “almost never,” 2 represents “sometimes,” 3 represents “often,” and 4 represents “most of the time.” Then, sum the values of these six questions.	0.437
	Attitude toward life	Based on the responses to the two questions in the CFPS questionnaire, namely, “How satisfied are you with your life?” and “How confident are you about your future?,” respondents are scored on a scale of 1 to 5. A score of 5 indicates “very dissatisfied” or “not confident at all,” and a score of 1 indicates “very satisfied” or “very confident.”	0.001

^1^Compared with standardized tools such as the World Health Organization’s Five Well-Being Index (WHO-5), this study uses the CFPS questionnaire to measure mental health comprehensively from two dimensions: mental state and life attitude. This approach encompasses multiple facets of psychological well-being, including emotional experiences, physical sensations, social experiences, and perceptions of life and the future—thus providing a more thorough coverage of different layers of mental health. It is well-suited for identifying potential psychological distress, offering a more complete psychological profile, and using language more aligned with local cultural descriptions of mental states. In contrast, standardized tools like the WHO-5 may focus narrowly on specific domains such as emotions and vitality, overlooking other factors that significantly impact mental health, such as confidence in the future.

#### 3.2.2 Independent variables

The independent variable in this paper is charitable medical care (charity care). Broadly speaking, charitable medical care includes not only tangible resources such as charitable donations but also intangible resources such as free medical consultations and services. Considering the availability of research data and that charitable donations are the main form of charitable medical care in China at present, this paper measures the explanatory variable by using the natural logarithm of the amount of charitable medical care donations received by the province where the residents are located. Due to the fragmented nature of charitable healthcare data and the lack of a unified database, research data were manually collected by the authors from relevant sources such as the Charity Blue Book: China Charity Development Report, China Statistical Yearbook, and China Civil Affairs Statistical Yearbook.

#### 3.2.3 Mediating variables

The mediating variables in this study are the accessibility of medical services (Acc - Med) and the quality of medical services (Qua - Med). According to relevant research, considering factors such as possible physical disabilities and individual needs, the perceptions of individual residents can better measure the accessibility and quality of medical services (56, 57). With respect to the accessibility of medical services, the response to the question “How satisfied are you with the conditions of accessing medical care?” in the CFPS questionnaire, scores are given on a scale of 1–5. A score of 1 represents “very dissatisfied,” and a score of 5 represents “very satisfied.” In the CFPS questionnaire, the definition of “medical care conditions” includes not only conditions related to doctors, medicine, diagnosis, hospitalization, etc., but also the distance to the medical facility and the convenience of transportation. Thus, a higher satisfaction score can indicate that the geographical and economic accessibility of medical treatment for residents is more easily met. To further enhance the reliability of this indicator, this study introduces the Health Resources Density Index (HRDI) proposed by Zheng and Ling (58) to measure the comprehensive level of resource distribution by population and geography in the region. The index is calculated as the geometric mean of health resources per 1,000 population and health resources per 1,000 square kilometers. This study constructs resource density indices corresponding to health institutions, hospital beds, and health technical personnel in residents’ provinces, and uses principal component analysis (PCA) to extract common factors. For the quality of medical services, based on the response to the question “What do you think of the medical level of the medical facility?” in the CFPS questionnaire, scores are given on a scale of 1–5. A score of 1 represents “very poor,” and a score of 5 represents “very good.”

#### 3.2.4 Control variables

Drawing on existing research, this paper selects the following control variables:

In terms of demographic characteristics:

(1) Age; (2) Gender, where 1 represents male and 0 represents female; (3) Household registration (Hukou), with 1 indicating nonagricultural household registration and 0 representing agricultural household registration; (4) Ethnicity, where 1 represents Han ethnicity and 0 represents other ethnic groups; (5) Marital status (Marry), with 1 denoting married and 0 signifying unmarried; (6) Years of education (Edu).

In terms of economic characteristics:

(1) Employment status (Employ), where 1 represents employed and 0 represents unemployed; (2) annual income (Inc), measured by the natural logarithm of annual income plus one.

In terms of health characteristics:

(1) Medical insurance (Ins), with 1 representing insured and 0 representing uninsured; (2) frequency of physical exercise (exercise).

Furthermore, at the provincial level, considering the potential relationship between provincial medical resource allocation and residents’ health and referring to relevant research, this paper selects four provincial-level control variables:

(1) The coefficient of population ageing (Old); (2) the number of medical and health institutions (Ins-Med), measured by the natural logarithm; (3) the number of health technicians per thousand population (Heal-Per); (4) the number of beds in medical and health institutions per thousand population (Bed-Med).

### 3.3 Descriptive statistics

Table 2 reports the descriptive statistical data of the key variables in this study, such as the mean, standard deviation, minimum value, median, and maximum value. Additionally, the variance inflation factor (VIF) is calculated to examine the issue of multicollinearity. In this study, the VIF values range from 1.01 to 4.54, with an average of 1.76, which is less than the common thresholds of 10 and 5 (59). This finding indicates that multicollinearity is not a major problem in this research.

**Table 2:** Descriptive Statistics (*N* = 49,593).

Variables	Mean	SD	Min.	Median	Max.
Health	−0.748	0.167	−0.999	−0.777	−0.115
P-Health	−0.201	0.067	−0.225	−0.225	−0.004
M-Health	−0.372	0.071	−0.438	−0.383	−0.110
S-Health	−0.175	0.098	−0.337	−0.169	0
Charity care	17.502	1.992	9.945	17.856	21.994
Age	54.065	21.251	18	51	120
Gender	0.525	0.499	0	1	1
Hukou	0.273	0.445	0	0	1
Ethnicity	0.990	0.099	0	1	1
Marry	0.840	0.366	0	1	1
Edu	5.187	5.784	0	0	22
Employ	0.816	0.388	0	1	1
Inc	1.629	1.110	0	2.353	2.842
Ins	0.886	0.318	0	1	1
Exercise	3.388	2.776	0	3	50
Old	10.676	2.423	4.980	9.940	17.420
Ins-Med	10.472	0.720	8.328	10.492	11.373
Heal-Per	5.865	1.303	3.580	5.700	12.610
Bed-Med	5.148	1.073	3.350	4.870	7.950

### 3.4 Model construction

In this study, the independent variable is charity care, which is measured by the natural logarithm of the number of charity care donations received by the province where the residents are located. This is a provincial-level variable. The dependent variable is the residents’ health level, which is an individual-level variable. Therefore, there may be a problem of data nesting in the research data; that is, individual-level data are nested within provincial-level data. If a traditional linear regression model is used at this time, due to the lack of independence among observations and the high within-group correlation, it may violate the assumption of sample independence in traditional regression, resulting in biased estimates. In this study, the health level of residents was affected not only by individual-level factors but also by factors such as the allocation of medical resources at the provincial level. Thus, there is a data-nesting problem, and using a multilevel regression model is more suitable for this study.

The multilevel regression model, also known as the hierarchical linear model (HLM) or the mixed-effects model, can consider multiple levels of data within a unified analytical framework and effectively analyze the variability between and within levels (60). Compared with the traditional linear regression model, the multilevel regression model can better handle the relationships among different levels in the research data, thus ensuring the validity of the estimation results. Therefore, this paper constructs the following model.

#### 3.4.1 Null model

In the null model, no independent variables or control variables are added. This is used to test whether there are provincial differences in the health levels of individual residents. Equation 1 represents a simple regression model equation for multilevel data:


(1) $$Y_{ij}=\beta +\varepsilon_{ij}$$

Equation 2 represents dividing the variance into two independent parts for estimation:


(2) $$\varepsilon_{ij}=\mu_{j}+e_{ij}$$

where, $\mu_{j}$ represents the between-group variance, and $e_{\mathrm{ij}}$ represents the within-group variance. Equation 3 represents the regression equation:


(3) $$Y_{ij}=\beta +\mu_{j}+e_{ij}$$

#### 3.4.2 Full model

Equation 4 represents the first-level model:


(4) $$Y_{ij}=\beta_{0j}+\beta_{1j}X_{\mathrm{ij}}+\varepsilon_{ij}$$

Equations 5 and 6 represent the second-level model:


(5) $$\beta_{0j}=\gamma_{00}+\gamma_{01}Z_{j}+\mu_{0j}$$

(6) $$\beta_{1j}=\gamma_{10}+\gamma_{11}Z_{j}+\mu_{1j}$$

Based on Equations 4–6, Equation 7 represents the complete model:


(7) $$Y_{ij}=(\gamma_{00}+\gamma_{01}Z_{j}+\mu_{0j})+(\gamma_{10}+\gamma_{11}Z_{j}+\mu_{1j})X_{\mathrm{ij}}+\varepsilon_{\mathrm{ij}}$$

where, $Y_{\mathrm{ij}}$ is the dependent variable of resident i in province j, $X_{\mathrm{ij}}$ is the independent variable of resident i in province j, $\beta_{0j}$ is the intercept term related to province j, $\beta_{1j}$ is the slope, $\varepsilon_{\mathrm{ij}}$ is the random error term, $Z_{j}$ represents the provincial-level variable, $\gamma_{00}$ and $\gamma_{10}$ are the intercepts of the fixed effects, $\gamma_{01}$ and $\gamma_{11}$ are the slopes of the fixed effects, and $\mu_{0j}$ and $\mu_{1j}$ are the random effects.

## 4 Empirical results and data analysis

### 4.1 Analysis results of the null model

The prerequisite for applying the multilevel regression model is to examine the proportion of the between-group variance in the total variance, that is, the intraclass correlation coefficient (ICC). The calculation formula for the null model is as follows:


(8) $$\mathrm{ICC}(\rho )=\frac{\sigma_{\mu_{j}}^{2}}{\sigma_{\mu_{j}}^{2}+\sigma_{e_{\mathrm{ij}}}^{2}}$$

According to Equation 8, the calculated ICC is 0.479, indicating that 47.9% of the total variation in health levels among individual residents is caused by differences at the provincial level. According to the criteria proposed by Cohen (1988) (61), when 0.01<=ρ<=0.059 , it represents a low-level association strength; when 0.059<=ρ<=0.138 , it represents a medium-level association strength; and when ρ>=0.138 , it represents a high-level association strength. When the ICC value is greater than 0.059, a multilevel regression model should be used. In this study, the ICC value is 0.479, indicating a high-level association strength, so the multilevel regression model is applicable.

### 4.2 Analysis results of the full model

Table 3 presents the regression results of the multilevel linear model based on Model (7). Column (1) presents the estimation results of the null model, and Column (2) presents the estimation results of the full model. Since this study inverted residents’ health indicators by taking their opposite values, as shown in Table 3, charitable medical care has a significant positive effect on the evaluation index of residents’ health level at the 1% significance level. This indicates that the more developed the charitable medical care is, the higher the score of the evaluation system is, and the higher the health level of local residents is.^9^ Moreover, charitable medical care can improve residents’ health levels in an all-round way, rather than having a single-aspect effect on physical or mental aspects. According to Table 3, the estimated coefficient of charitable medical care on residents’ health level is 0.005, which means that for every one-unit increase in charitable medical care, the health level of residents increases by 0.005 units. Compared with the average score of −0.748 for overall health, this result indicates that charitable healthcare increases residents’ overall health scores by 0.668%—a change that is economically significant. In conclusion, Research Hypothesis 1 of this paper is confirmed.

**Table 3:** Regression Results of the Multilevel Linear Model.

Variables	Model 0 (1)	Model 1 (2)
Fixed effects section		
Charity Care		0.0 (0.001)
Age		0.00 (0.001)
Gender		−0.034 (0.002)
Hukou		−0.0 (0.002)
Ethnicity		0.029** (0.007)
Marry		0.048*** (0.002)
Edu		0.005*** (0.001)
Employ		−0.025*** (0.002)
Inc		−0.011*** (0.001)
Ins		0.011*** (0.002)
Exercise		−0.004*** (0.001)
Old		−0.004*** (0.001)
Ins-Med		−0.018*** (0.001)
Heal-Per		−0.011*** (0.001)
Bed-Med		0.019*** (0.001)
Random effects section		
Sigma_u	0.116 (0.001)	0.104 (0.001)
Sigma_e	0.121 (0.001)	0.121 (0.001)
cons	−0.746*** (0.001)	−0.748*** (0.017)
N	49,593	49,593

*, **, and *** represent significance at the 10, 5, and 1% levels, respectively. Standard errors are in parentheses. The same applies to the following table.

### 4.3 Endogeneity test

#### 4.3.1 Instrumental variable (2SLS)

To address potential endogeneity issues such as bidirectional causality and avoid bias in the estimation results, this study employs the instrumental variable method (2SLS) for endogeneity testing. Drawing on existing research (62), we select the number of religious venues in residents’ provinces (Religion)^10^ as an instrumental variable for charitable healthcare and conduct 2SLS regressions. This instrumental variable is chosen for two reasons. First, there is a correlation between the number of religious venues and charitable healthcare. Religious cultures often advocate values such as compassion, mercy, and aiding others, which may subtly influence people to engage in more charitable activities. As a result, provinces with more religious venues tend to have a stronger religious cultural atmosphere, making residents more likely to be influenced by religious values and thus inclined to participate in more charitable healthcare initiatives. Second, the number of religious venues is uncorrelated with the error term and does not directly affect residents’ health status, thus qualifying it as a strictly exogenous variable. Taken together, this variable meets the requirements for instrumental variable relevance and exogeneity.

According to columns (1)–(2) in Table 4, in the first-stage instrumental variable regression, the instrumental variable is significantly correlated with the core explanatory variable at the 1% significance level. The *F*-value of 732.91 exceeds 10, indicating that the weak instrument test is passed. The Kleibergen-Paap rk LM test yields a *p*-value below 0.1, rejecting the null hypothesis of instrumental variable under-identification. The Cragg-Donald Wald F-value of 732.91 is significantly greater than the 10% critical value of 16.38, rejecting the null hypothesis of weak instrumental variable correlation. In the second-stage instrumental variable regression, the estimated coefficient of the core explanatory variable remains significantly positive at the 1% level, and its magnitude is comparable to that in the benchmark regression. Thus, the research conclusion aligns with previous findings.

**Table 4:** Regression results of the instrumental variables.

Variables	Charity care (1)	Health (2)
Charity care		0.010*** (0.003)
Religion	0.242*** (0.009)	
Kleibergen–Paap rk Wald F	732.91 > 10	
Kleibergen–Paap rk LM	722.27 (P-val = 0.0000)	
Cragg-Donald Wald F	732.91 > 16.38	
Controls	Yes	Yes
*N*	49,593	49,593

#### 4.3.2 Heckman two-stage regression

To address endogeneity issues arising from sample selection bias, this paper uses the Heckman two-stage regression model. In the first-stage regression, observations with charitable medical donations received above the median are classified as the experimental group (Don_dum), and assigned a value of 1. Those below or at the median are assigned 0. In the first stage, the instrumental variable (Religion) described earlier is used for Probit regression to calculate the Inverse Mills Ratio (IMR). The IMR is then included in the second-stage regression model. As shown in column (1) of Table 5, the regression coefficient of the explanatory variable is significantly positive at the 1% significance level. Column (2) shows that the estimated IMR coefficient is insignificant, while the estimated coefficient of charitable healthcare on residents’ health remains significantly positive at the 1% level. Thus, the research sample does not exhibit obvious sample selection bias.

**Table 5:** Heckman two-stage regression results.

Variables	Don_dum (1)	Health (2)
Charity care		0.005*** (0.001)
Religion	0.234*** (0.009)	
IMR		0.005 (0.004)
Controls	Yes	Yes
cons	−14.966*** (0.160)	−0.802*** (0.044)
*N*	49,593	49,593
Pseudo R^2^ / R-sq	0.296	0.088

### 4.4 Robustness test

#### 4.4.1 Replacement of core variables

To further ensure the robustness of the research conclusions, this paper conducts regression analysis again by replacing the core variables. With respect to the dependent variable, this paper uses the response to the question “Have you suffered from a doctor-diagnosed chronic disease in the past six months?” in the CFPS questionnaire as an alternative variable (Dis) for health level. A value of 1 indicates “yes,” and 0 indicates “no.” For the independent variable, this paper uses the proportion of the charitable donation amount received by each province in the total national charitable donation amount (Pro - Charity) as a replacement. After replacing the core variables, this paper substitutes them into Model (7) for regression analysis again. The results are shown in Table 6. When both the dependent and independent variables in this paper are replaced, the estimated coefficient is significantly negative, which is consistent with the previous conclusions. This proves the robustness of the empirical results of this study.

**Table 6:** Replacement of core variables.

Variables	Dis
Fixed effects section	
Pro-charity	−0.399*** (0.021)
Control variables	Yes
Random effects section	
Sigma_u	0.141 (0.003)
Sigma_e	0.275 (0.001)
_cons	0.985*** (0.031)
N	49,593

#### 4.4.2 Elimination of the impact of sudden events

This paper selects sample data from 20,122,020. Among them, the COVID-19 pandemic in 2020 might have had a significant effect on the overall health level of residents, leading to biases in the estimation results. Therefore, to mitigate the impact of sudden events on the regression results, this paper excludes the samples from 2020 from the sample data and conducts the regression again. According to Table 7, after excluding the observations from 2020, the estimated coefficients are still significantly positive at the 1% level, and the estimation results are similar to those in the previous section. This finding indicates that sudden events have not had a significant effect on the results of the empirical analysis, thus demonstrating the reliability of the analysis in this paper.

**Table 7:** Elimination of the impact of sudden events.

Variables	Health
Fixed effects section	
Charity care	0.007*** (0.001)
Control variables	Yes
Random effects section	
Sigma_u	0.102 (0.001)
Sigma_e	0.126 (0.001)
_cons	−0.769*** (0.019)
*N*	41,859

#### 4.4.3 Exclusion of samples from municipalities directly under the central government

In China, the four municipalities directly under the Central Government, namely, Beijing, Tianjin, Shanghai, and Chongqing, usually have large built-up areas and large resident populations. Moreover, they enjoy obvious advantages in terms of national politics, economy, science, transportation, culture, income, etc. The health levels of their residents may differ significantly from those of other provinces. Therefore, this study excludes the research samples from these four municipalities and substitutes them into Model (7) for regression analysis again. The analysis results are shown in Table 8. According to Table 8, the regression results are consistent with the previous results, which proves the robustness of the conclusions in this paper.

**Table 8:** Exclusion of samples from municipalities directly under the central government.

Variables	Health
Fixed effects section	
Charity care	0.005*** (0.001)
Control variables	Yes
Random effects section	
Sigma_u	0.105 (0.001)
Sigma_e	0.122 (0.001)
_cons	−0.679*** (0.023)
*N*	44,934

### 4.5 Test results of the mechanism variables

The previous empirical test results indicate that the development of charitable medical care significantly promotes improvements in residents’ health levels. Next, this paper refers to the general practice of mediating effect testing in the academic community (41) and analyses the possible action paths according to Model (7). In this study, the dependent variable of Model (7) is replaced with medical service accessibility and medical service quality to test whether they are the transmission mechanisms through which charitable medical care affects residents’ health levels. The test results are shown in Table 9. Among these, the accessibility of medical services (Acc - Med) is jointly measured by two indicators: “satisfaction with the conditions of accessing medical care” (Satis) and the health resource density index (HRDI), capturing both subjective and objective dimensions. Column (1) and Column (2) of Table 9 shows the results of the mediating analysis of medical service accessibility, and Column (3) shows the results of the mediating analysis of medical service quality. According to Column (1) and Column (2), the estimated coefficients of charitable medical care on medical service accessibility are 0.037 and 0.004, and it is significantly positive at the 1% level, an increase in the amount of charitable medical donations enhances residents’ satisfaction with the conditions of accessing medical care and the local health resource density, thereby enabling residents to more easily access and utilize medical resources. According to Column (3), the estimated coefficient of charitable medical care on medical service quality is 0.028, and it is significantly positive at the 1% level, indicating that more charitable medical donations can promote the improvement of medical service quality. Combined with the previous theoretical analysis, this study suggests that medical service accessibility and medical service quality play a mediating role, and the transmission paths are as follows: “Charitable medical care → Accessibility of medical services → Residents’ health” and “Charitable medical care → Quality of medical services → Residents’ health.”

**Table 9:** Regression results of mechanism variables.

Variables	Satis	HRDI	Qua - Med
	(1)	(2)	(3)
Fixed effects section			
Charity care	0.037*** (0.003)	0.004*** (0.001)	0.028*** (0.003)
Control variables	Yes	Yes	Yes
Random effects section			
Sigma_u	0.425 (0.068)	1.281 (0.163)	0.311 (0.046)
Sigma_e	0.900 (0.003)	0.438 (0.001)	0.941 (0.003)
_cons	7.643*** (0.834)	−2.080*** (0.233)	5.566*** (0.621)
*N*	49,593	49,593	49,593

To further verify the robustness of the regression results of the mechanism test, this paper uses the bootstrap test method to analyze and test the action paths again. The bootstrap test method can repeatedly sample from the research sample (in this paper, the number of repeated samples is set to 1,000). It directly tests the significance of the product of coefficients and is highly persuasive for verifying the possible mediating effects. The statistical results are shown in Table 10. Under the 95% confidence interval, the confidence intervals of each mechanism variable do not contain 0, which indicates that the mediating effects are significant. Therefore, combined with the theoretical analysis, Hypothesis 2 of this paper is verified.

**Table 10:** Bootstrap Test.

Mediating Variables		Effect	Boot SE	Boot LLCI	Boot ULCI
Acc - Med	Satis	0.007***	0.001	−0.010	−0.0045
	HRDI	0.004***	0.001	−0.005	−0.002
Qua - Med		0.004***	0.001	−0.006	−0.001

## 5 Further analysis

### 5.1 Heterogeneity analysis based on residents’ trust

Charity is a product of society, rooted in the spirit of humanism and altruism, and driven by individuals’ moral codes and volunteer spirit. As a social-psychological phenomenon, the degree of residents’ trust in others may affect the effectiveness of charitable medical care in improving residents’ health levels. Therefore, in response to the question “Do you prefer to trust others or suspect others?” in the CFPS questionnaire, this paper divides the observation samples into two groups: high-trust and low-trust groups for heterogeneity analysis. The analysis results are shown in Table 11. For both groups, charitable medical care has a significantly positive effect on the score of residents’ health at the 1% level. However, for the group of residents with high levels of trust, the promoting effect of charitable medical care on the health level is greater than that of the low-trust group. The possible reasons are as follows. First, in a highly trusted social environment, residents have a high degree of trust in charitable medical projects, which encourages them to be more willing to participate in relevant charitable medical activities. Second, the greater the degree of residents’ trust is, the greater their willingness to accept charitable medical services. Moreover, it helps medical institutions allocate and utilize charitable resources more effectively, ensuring the fairness of medical services. Finally, in a highly trusted social environment, residents are more inclined to believe in the quality and effectiveness of charitable medical care, thus indirectly promoting improvements in health levels. In contrast, in a social environment with low levels of trust, residents may be sceptical about charitable causes, which negatively affects the acceptance of charitable medical care.

**Table 11:** Heterogeneity Analysis Based on Residents’ Trust.

Variables	High - trust	Low - trust
	(1)	(2)
Fixed effects section		
Charity care	0.006*** (0.001)	0.004*** (0.001)
Control variables	Yes	Yes
Random effects section		
Sigma_u	0.108 (0.002)	0.102 (0.001)
Sigma_e	0.129 (0.001)	0.113 (0.001)
_cons	−0.741*** (0.025)	−0.783*** (0.021)
N	21,913	27,680

### 5.2 Heterogeneity analysis based on family income levels

Charitable medical care is a social supplementary mechanism to ensure that economically disadvantaged groups and those in remote and underdeveloped areas have access to basic medical services. Therefore, as annual family income increases, the impact of charitable medical care on residents’ health levels may decrease. To verify this issue, this paper divides the observation samples into three groups—low-income, middle-income, and high-income groups—on the basis of their responses to the question about total family income in the CFPS questionnaire and conducts a heterogeneity analysis. The analysis results are shown in Table 12. As the annual family income increases, the estimated coefficients of the impact of charitable medical care on residents’ health gradually decrease, and all the coefficients are significant at the 1% level. This finding indicates that the impact of charitable medical care on residents’ health levels weakens as annual family income increases. This proves that as the annual family income increases, the economic strength of residents significantly increases. They can cope with potential medical risks by purchasing commercial health insurance and increasing their ability to pay for medical expenses out of pocket. This self-sufficiency trend weakens residents’ dependence on and demand for charitable medical care.

**Table 12:** Heterogeneity analysis based on family income levels.

Variables	High	Middle	Low
	(1)	(2)	(3)
Fixed effects section			
Charity care	0.002*** (0.001)	0.003*** (0.001)	0.011*** (0.001)
Control variables	Yes	Yes	Yes
Random effects section			
Sigma_u	0.089 (0.001)	0.097 (0.002)	0.107 (0.002)
Sigma_e	0.104 (0.001)	0.122 (0.001)	0.138 (0.002)
_cons	−0.606*** (0.078)	−0.375*** (0.042)	−0.807*** (0.033)
N	16,530	16,530	16,533

### 5.3 Heterogeneity analysis based on total medical expenses

Against the dual backdrop of continuously rising medical costs and the intensifying trend of population ageing, the growth of residents’ total medical expenses has become a popular issue that society is closely observing. In this context, as an important part of the multilevel medical security system, charitable medical care provides necessary medical assistance to residents who are experiencing financial difficulties and have urgent medical needs. This paper divides the samples into two groups, the low- expense group and the high- expense group based on the responses to the question “total medical expenses” in the CFPS questionnaire, and conducts heterogeneity analysis. The analysis results are shown in Table 13. Compared with the low-expense group, for the high- expense group, the estimated coefficient of the impact of charitable medical care on residents’ health levels is larger, and it is significant at the 1% level. This finding indicates that the promoting effect of charitable medical care on residents’ health levels is more effective among the high-expense group. The possible reason is the group with high total medical expenses usually experiences more serious disease or health problems and requires more medical services and resources. These groups often face financial difficulties due to high medical expenses and may even be pushed into poverty because of illness. Intervention in charitable medical care can significantly reduce their financial burden, enabling them to access necessary medical services and thus more effectively promoting improvements in their health levels.

**Table 13:** Heterogeneity analysis based on total medical expenses.

Variables	Low - expense	High - expense
	(1)	(2)
Fixed effects section		
Charity care	0.0055*** (0.001)	0.0057*** (0.001)
Control variables	Yes	Yes
Random effects section		
Sigma_u	0.075 (0.001)	0.105 (0.002)
Sigma_e	0.098 (0.001)	0.138 (0.001)
_cons	−0.804*** (0.017)	−0.722*** (0.026)
N	26,636	22,957

## 6 Summary and discussion

### 6.1 Research conclusions

This paper uses survey data from five years, namely, 2012, 2014, 2016, 2018, and 2020, of the CFPS to form panel data. Using the entropy weight method, an evaluation index system for residents’ health levels is constructed. It then uses a multilevel regression model to empirically analyze the relationship between charitable medical care and residents’ health levels, as well as the mediating effects of medical service accessibility and medical service quality. The research findings are as follows: First, an increase in the amount of charitable medical donations significantly promotes improvements in residents’ health levels. This result still holds even after addressing endogeneity via the instrumental variable approach and Heckman two-stage regression, and conducting robustness tests such as replacing core variables, excluding the impact of emergencies, and removing samples from municipalities directly under the Central Government. Second, this paper proves that medical service quality and medical service accessibility are the paths through which charitable medical care affects residents’ health levels. That is, the development of charitable medical care will promote improvements in medical service accessibility and medical service quality, thereby improving residents’ health levels. This conclusion still holds after being tested by the bootstrap method. Finally, in the heterogeneity test section, this study revealed that, for residents with higher levels of trust, lower annual family income, and higher total medical expenses, the promoting effect of charitable medical care on residents’ health levels is stronger.

### 6.2 Policy recommendations

This study confirmed that charitable medical care plays a significant role in promoting improvements in residents’ health levels. Currently, China is in a crucial period of comprehensively advancing the “Healthy China” strategy. With the accelerating process of population ageing and increasing disease burden, the medical and health fields are facing structural contradictions, such as uneven distributions of medical resources and limited scopes of medical insurance reimbursement. Against this background, accelerating the development of charitable medical care is not only a beneficial supplement to the multilevel medical security system but also an important practical path for building a national health security network. First, improve the incentive mechanism and supervision system for charitable medical donations. Relevant government departments should improve and introduce more refined incentive policies for charitable donations, including tax credits and matching donations, to encourage the flow of social resources into the field of charitable medical care. Moreover, individuals or organizations that have made outstanding contributions in the field of charitable medical care should be given appropriate honor and recognition to enhance the overall charitable atmosphere in society. In addition, a sound and transparent supervision framework for charitable medical donations should be established to ensure the rational allocation and use of charitable resources. For example, improve the mechanism for making charitable medical donations public and transparent, regularly announce the sources, destinations, and usage of donation funds strengthen the auditing and evaluation of charitable organizations and medical institutions to ensure that they comply with relevant laws and regulations and operate in a standardized manner.

Second, promote the in-depth integration of charitable medical care and the public health system. Explore and establish a collaborative mechanism between charitable medical care and the public health system. Through methods such as information sharing and resource complementation, increase the overall efficiency of medical services. A digital information docking platform between charitable medical care and the public health system should be promoted and established to achieve real-time sharing and optimal allocation of medical resources, ensuring that charitable medical resources can be allocated precisely to the resident groups most in need of help. Moreover, encourage charitable organizations to establish cooperative relationships with medical institutions such as public hospitals and community health service centres to carry out activities such as medical assistance and health education jointly.

Third, establish a –long-term evaluation system for charitable medical care. Develop a comprehensive and systematic evaluation mechanism to continuously track the changing trends of charitable medical donations, the fairness of medical services, the quality of medical services, and the health levels of residents. Regularly collect, organize, and analyze relevant data regularly evaluate the implementation effects of charitable medical care and assess its economic and social benefits. In addition, in-depth cooperation and exchanges with international charitable organizations should be strengthened to learn from foreign advanced experiences and continuously improve China’s charitable medical system.

Fourth, strengthen the publicity of charitable medical care. Use traditional media such as television, radio, and newspapers to widely publicize the advantages, characteristics, and relevant policies of charitable medical care. Leverage new media channels such as social media platforms and short video platforms to improve the dissemination speed and interactivity of information. Moreover, strengthen the cooperative relationships among medical institutions, enterprises, schools, and communities, and use their well-established network systems to promote charitable medical projects. According to the characteristics and needs of different target audiences, publicity materials that are easy to understand, such as posters, brochures, and short videos, can be designed to increase residents’ trust in and support charitable medical projects.

Fifth, establish a “charitable healthcare + commercial insurance” multi-tiered protection model to avoid a “dependence trap.” When developing charitable healthcare, it is crucial to recognize that when not combined with individual responsibility, pure charitable relief may lead to “welfare dependence,” eroding society’s collective sense of risk sharing and potentially crowding out market mechanisms with charitable resources. Charitable healthcare should focus on emergency relief and critical illness support — such as rare disease treatments and one-time grants for severe illnesses — while avoiding coverage of routine outpatient care, chronic disease management, and other areas that can be addressed by insurance. Governments and charitable organizations can guide low-income groups to purchase inclusive commercial insurance via premium subsidies, tax incentives, and other measures. Meanwhile, setting eligibility criteria or time limits for charitable healthcare can encourage recipients to transition to insurance-based coverage as their economic situation improves, preventing “lifelong dependence” and ensuring the sustainability and fairness of charitable healthcare.

## 7 Limitations and future research

On the one hand, this study fails to empirically examine the impact of charitable medical care on residents’ health in a broad sense. In China, charitable medical care encompasses not only charitable donations of tangible resources such as funds, drugs, and medical devices but also intangible resources such as free medical consultations for specific groups or diseases, policy promotion, and expertise and technology. Given that charitable donations are the primary form of current charitable medical care and that there is a severe shortage of relevant data on intangible resources such as free medical consultations in each province, making it extremely difficult to collect such data, it is challenging to empirically test the health effects of charitable medical care. Therefore, this paper focuses on the impact and mechanism of charitable medical donations on residents’ health. On the other hand, data related to charitable medical donations at the individual level are lacking. In this study, the dependent variable is the data on charitable medical donations at the provincial level, whereas the independent variable is the data on health levels at the individual level. This research constructed a multilevel regression model and conducted a series of robustness and endogeneity tests to address the problem of ecological fallacy in the research data. Owing to the current serious shortage of relevant charitable data at the individual level in China and the lack of an established large-scale database, this paper failed to collect data related to individual-level charitable medical donations in all provinces across the country. In summary, the lack of exploration of intangible resources may lead to bias in effect estimation, thereby failing to fully reflect the synergistic effects of various resources in charitable healthcare on residents’ health. In terms of generalizability, as this study focuses on charitable donations and does not cover all mechanisms through which charitable healthcare influences residents’ health, it limits the development of a theoretical framework with broad explanatory power. Future research could attempt to integrate data from multiple stakeholders—governments, charitable organizations, and healthcare institutions—to provide more comprehensive data support for the analysis.

## Data Availability

Publicly available datasets were analyzed in this study. This data can be found at: https://cfpsdata.pku.edu.cn/#/home, China Family Panel Studies (CFPS).

## Footnotes

^1^The fundamental goal of the “Healthy China” strategy is to achieve universal health. The strategic objectives are as follows: by 2020, the major health indicators should rank among the top in middle - and high - income countries; by 2030, the major health indicators should enter the ranks of high - income countries; and by 2050, a healthy country commensurate with a modern socialist country should be established. For detailed indicators, please refer to https://www.gov.cn/zhengce/202203/content_3635233.htm.

^2^For the full content of the report, please visit https://www.gov.cn/xinwen/2022-10/25/content_5721685.htm.

^3^The data is sourced from the “Statistical Communiqué on the 2023 National Economic and Social Development of the People’s Republic of China” released by the National Bureau of Statistics of China.

^4^This report is the first volume of “China Social Public Welfare and Charity Guide,” released by the Center for Asian Philanthropy and Society (CAPS). For detailed content, please refer to http://caps.org.

^5^Online platforms such as Shuidichou and Qingsongchou are typical crowdfunding platforms for major - illness medical care that have emerged in China, relying on internet technology. They aim to help individuals and families who are in financial distress due to illness or accident raise medical expenses quickly, representing an innovative form of civil mutual assistance in the internet age.

^6^The CFPS (China Family Panel Studies) focuses on the economic and non - economic well - being of Chinese residents, as well as numerous research topics including economic activities, educational achievements, family relationships and family dynamics, population migration, health, etc. It is a national - scale, large - scale, multi - disciplinary social tracking survey project.

^7^The CSMAR database is an economic and financial database developed by drawing on the professional standards of internationally renowned databases such as CRSP of the University of Chicago, Standard & Poor’s Compustat, TAQ of the New York Stock Exchange, and ISDA, and integrating China’s actual national conditions. This database covers 19 major series including personal characteristics, banking research, stock market, and macro - economy, containing more than 200 databases, over 4,000 tables, and more than 60,000 fields.

^8^Since all the evaluation indicators in the residents’ health assessment index system are negative indicators, the lower the final score, the higher the residents’ health level. For ease of understanding and interpretation, this paper transforms residential health indicators by taking their opposite values.

^9^Due to space limitations, since overall health is a comprehensive indicator for evaluating residents’ health levels, this paper does not present the regression results of physical health, mental health, and self - rated health. Instead, it uses overall health as a representative, and the same approach will be applied to the subsequent research results.

^10^The number of religious venues in each province was manually collected by this study from sources such as the Religion Blue Book: China Religion Report.
